# Supplementary material for: Fabrication, Optimization, and Characterization of Antibacterial Electrospun Shellac Fibers Loaded with Kaempferia parviflora Extract
Source: Pharmaceutics. 2022 Dec 29;15(1):123. doi: 10.3390/pharmaceutics15010123 (PMC9861391; doi:10.3390/pharmaceutics15010123)
Supplement: Supplementary file 1 [file pharmaceutics-15-00123-s001.zip › pharmaceutics-2052959-supplementary.pdf]

pharmaceutics

Supplementary Materials: Fabrication, Optimization, and Characterization of Antibacterial Electrospun Shellac Fibers Loaded with *Kaempferia parviflora* Extract

Wantanwa Krongrawa, Sontaya Limmatvapirat, Mont Kumpugdee Vollrath, Prasat Kittakoop, Supachai Saibua and Chutima Limmatvapirat

Table S1. Experimental design matrix and corresponding responses based on experimental runs proposed by BBD design.

Run	Factor 1 (x_1)	Factor 2 (x_2)	Factor 3 (x_3)	Factor 4 (x_4)	Response 1	Response 2
	Shellac concentration (% *w*/*w*)	Feed rate (mL/h)	Electrical voltage (kV)	Extract concentration (% *w*/*w*)	Fiber diameters (nm), *n* = 3 Mean ± SD	Bead-to-fiber ratio, *n* = 3 Mean ± SD
1	38	0.8	18	3	832 ± 6.07	0.24 ± 0.06
2	38	0.8	24	1	824 ± 7.56	0.32 ± 0.07
3	38	0.4	18	1	635 ± 8.40	0.41 ± 0.03
4	36	0.8	18	5	724 ± 8.88	0.42 ± 0.01
5	36	1.2	18	3	495 ± 9.14	0.58 ± 0.02
6	40	0.4	18	3	915 ± 9.23	0.04 ± 0.03
7	38	0.8	12	1	606 ± 9.20	0.30 ± 0.02
8	40	0.8	18	1	819 ± 9.06	0.14 ± 0.01
9	40	0.8	18	5	1149 ± 16.65	0.07 ± 0.01
10	40	0.8	24	3	962 ± 9.29	0.12 ± 0.02
11	40	0.8	12	3	864 ± 12.72	0.10 ± 0.01
12	38	0.4	24	3	807 ± 5.81	0.26 ± 0.02
13	40	1.2	18	3	878 ± 6.92	0.08 ± 0.00
14	38	0.8	18	3	766 ± 8.11	0.27 ± 0.03
15	36	0.4	18	3	475 ± 9.12	0.42 ± 0.06
16	38	1.2	18	1	473 ± 3.02	0.23 ± 0.04
17	38	1.2	24	3	788 ± 6.94	0.11 ± 0.02
18	38	1.2	18	5	954 ± 12.20	0.15 ± 0.03
19	38	1.2	12	3	728 ± 10.21	0.25 ± 0.04
20	38	0.4	18	5	1015 ± 29.34	0.28 ± 0.02
21	38	0.8	24	5	902 ± 25.32	0.21 ± 0.04
22	36	0.8	18	1	433 ± 4.02	0.86 ± 0.02
23	38	0.8	18	3	785 ± 4.86	0.30 ± 0.03
24	36	0.8	24	3	527 ± 7.43	0.81 ± 0.05
25	36	0.8	12	3	524 ± 8.83	0.78 ± 0.06
26	38	0.8	12	5	545 ± 9.19	0.29 ± 0.02
27	38	0.4	12	3	667 ± 14.45	0.38 ± 0.04

Table S2. ANOVA results for the prediction of fiber diameter and bead-to-fiber ratio of electrospun shellac fibers using the BBD matrix.

Source	Coefficients	*p*-value	Standard deviation	% CV	Adequate precision	R^2	F-value
Y_1 (diameter)			89.83	12.07	18.836	0.7983	
x_1	200.66	< 0.0001					
x_2	72.96	0.0099					
x_4	124.97	< 0.0001					
Intercept	744.07						
Model		< 0.0001					30.34
Lack of fit		0.1245					7.47
Y_2 (bead-to-fiber ratio)			0.11	35.76	18.598	0.7652	
x_1	-0.28	< 0.0001					
x_4	-0.07	0.0408					
Intercept	0.31						
Model		< 0.0001					39.10
Lack of fit		0.0643					14.99

Table S3. Verification of experimental results in relation to the predicted values generated by the designed models.

Run	Fiber diameters (nm)			Bead-to-fiber ratios		
	Predictive value	Acceptable interval (95% confident level)	Experimental value	Predictive value	Acceptable interval (95% confident level)	Experimental value
1	574.71	504.83-633.30	582.00	0.34	0.10-0.57	0.43
2			589.00			0.29
3			573.00			0.33

Table S4. Hot-stage microscope (HSM) images of shellac flakes and fibers at elevated temperatures.

**Temperature (°C)**	**HSM images**	
	**Shellac flakes**	**Shellac fibers**
50	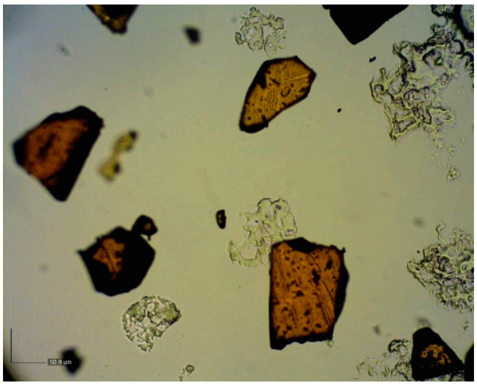	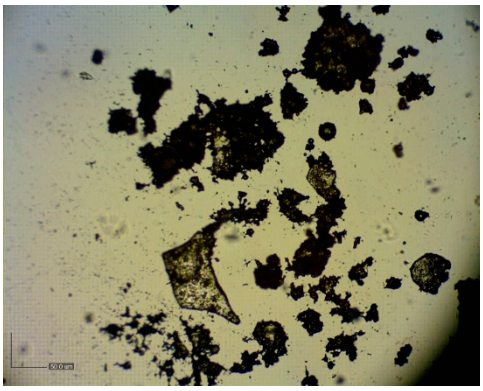
60	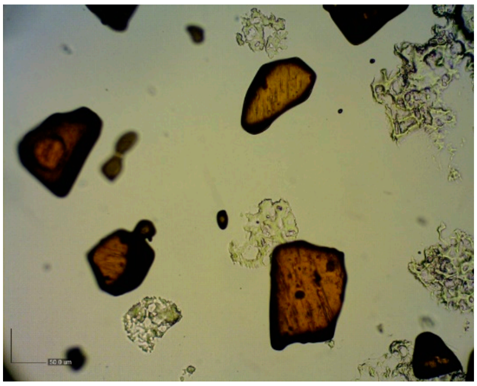	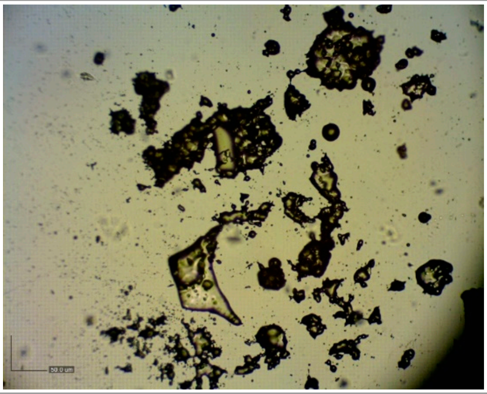
70	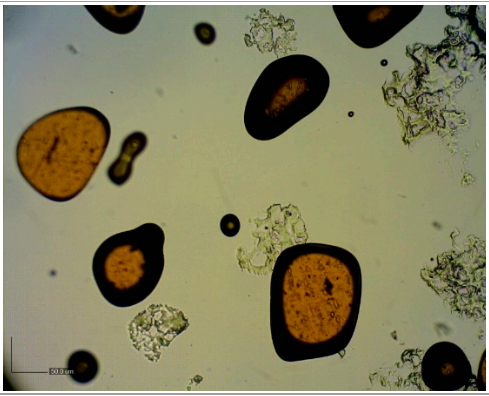	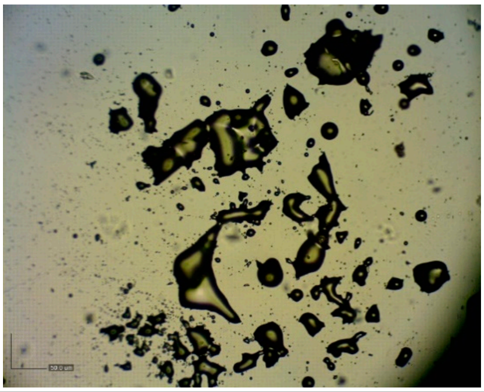

Table S5. Total methoxyflavones release kinetics models of KP extract-loaded electrospun shellac fibers with various formulations.

Modelling	Formulation	Parameters	R^2 Adjusted	AIC	MSC
Zero-order	36% (*w/w*) Shellac (Run 5)	k_0 = 0.658	0.6261	33.9314	0.5837
	38% (*w/w*) Shellac (Run 1)	k_0 = 0.362	0.8586	29.3385	1.5559
	40% (*w/w*) Shellac (Run 10)	k_0 = 0.279	0.7723	23.8660	1.0799
	1% (*w/w*) KP extract (Run 8)	k_0 = 0.707	0.7320	32.7120	0.9168
	3% (*w/w*) KP extract (Run 13)	k_0 = 0.717	0.9303	27.1213	2.2633
	5% (*w/w*) KP extract (Run 9)	k_0 = 0.548	0.1348	34.5053	-0.2552
	Run optimized	k_0 = 0.615	0.7643	31.5195	1.0452
First-order	36% (*w/w*) Shellac (Run 5)	k_1 = 0.011	0.8456	30.0709	1.3558
	38% (*w/w*) Shellac (Run 1)	k_1 = 0.005	0.8301	30.8168	1.2602
	40% (*w/w*) Shellac (Run 10)	k_1 = 0.003	0.8474	21.8649	1.4802
	1% (*w/w*) KP extract (Run 8)	k_1 = 0.012	0.9643	22.6377	2.9317
	3% (*w/w*) KP extract (Run 13)	k_1 = 0.012	0.9413	25.6829	2.3226
	5% (*w/w*) KP extract (Run 9)	k_1 = 0.008	0.6404	30.1149	0.6228
	Run optimized	k_1 = 0.009	0.9037	27.6057	1.8279
Higuchi	36% (*w/w*) Shellac (Run 5)	k_H = 6.166	0.8683	28.7122	1.6275
	38% (*w/w*) Shellac (Run 1)	k_H = 3.948	0.7251	32.6617	0.8913
	40% (*w/w*) Shellac (Run 10)	k_H = 2.594	0.8548	21.6160	1.5299
	1% (*w/w*) KP extract (Run 8)	k_H = 6.605	0.9303	25.9781	2.2336
	3% (*w/w*) KP extract (Run 13)	k_H = 6.606	0.9096	28.4231	2.0030
	5% (*w/w*) KP extract (Run 9)	k_H = 5.224	0.8553	25.5361	1.5332
	Run optimized	k_H = 5.724	0.8888	27.7630	1.7965
Kosmeyer-Peppas	**36% (*w/w*) Shellac (Run 5)**	**k_{KP} = 4.217**<br>**n = 0.698**	**0.8995**	**27.3629**	**1.8974**
	**38% (*w/w*) Shellac (Run 1)**	**k_{KP} = 0.830**<br>**n = 0.612**	**0.8859**	**28.2652**	**1.7706**
	**40% (*w/w*) Shellac (Run 10)**	**k_{KP} = 1.242**<br>**n = 0.669**	**0.8684**	**21.6875**	**1.5156**
	**1% (*w/w*) KP extract (Run 8)**	**k_{KP} = 4.060**<br>**n = 0.612**	**0.9643**	**20.8333**	**3.5210**
	**3% (*w/w*) KP extract (Run 13)**	**k_{KP} = 2.245**<br>**n = 0.747**	**0.9957**	**13.7837**	**4.9309**
	**5% (*w/w*) KP extract (Run 9)**	**k_{KP} = 6.387**<br>**n = 0.554**	**0.9815**	**13.2558**	**1.1850**
	**Run optimized**	**k_{KP} = 3.012**<br>**n = 0.647**	**0.9519**	**23.5706**	**2.6350**

Note: The significance of **bold** reveals the estimated parameters derived from the best-fit mathematical model.

Table S6. Antimicrobial activity of KP extract-loaded electrospun shellac fibers as determined by time-kill kinetics assay.

		Time (min)		
		15	30	60
	Digital image	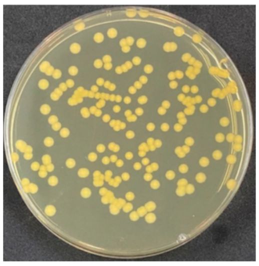	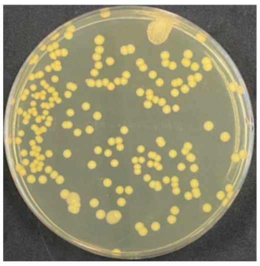	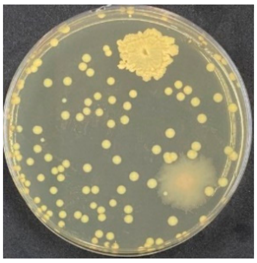
	Microbial load (CFU/mL)	1.09×10^{6}	1.87×10^{6}	1.18×10^{6}
	R (%)	-	-	-
S. aureus		Time (min)		
		120	240	360
	Digital image	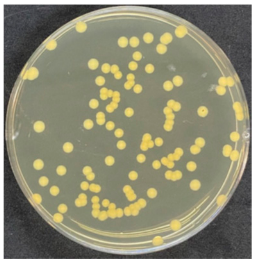	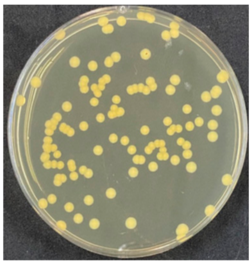	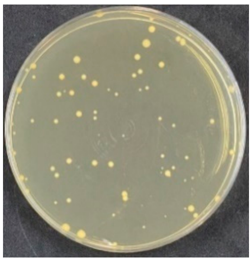
	Microbial load (CFU/mL)	7.80×10^{5}	1.60×10^{5}	1.30×10^{5}
	R (%)	28.44	85.32	88.07
		Time (min)		
		15	30	60
	Digital image	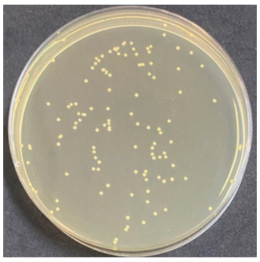	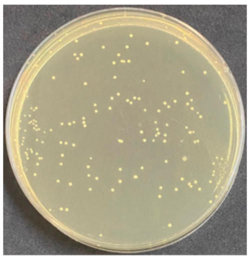	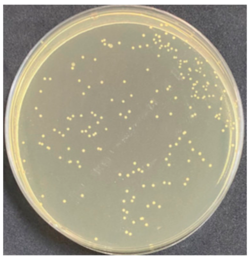
	Microbial load (CFU/mL)	1.09×10^{6}	1.62×10^{6}	2.62×10^{6}
	R (%)	-	-	-
E. coli		Time (min)		
		120	240	360
	Digital image	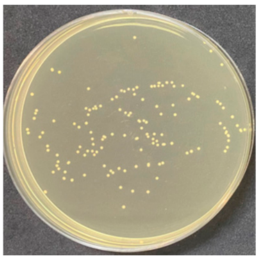	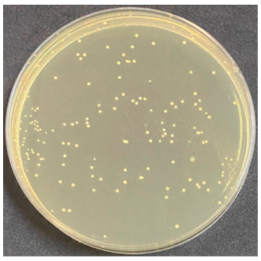	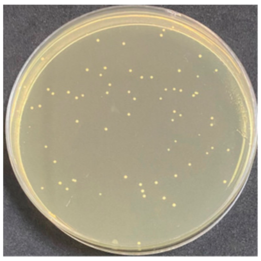
	Microbial load (CFU/mL)	1.34×10^{6}	9.99×10^{5}	8.60×10^{5}
	R (%)	-	9.17	21.10

Table S6 (continue). Antimicrobial activity of KP extract-loaded electrospun shellac fibers as determined by time-kill kinetics assay.

			**Time (min)**	
		15	30	60
	Digital image	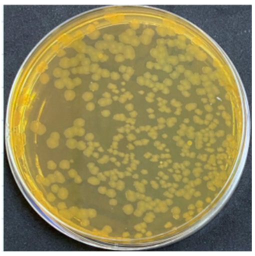	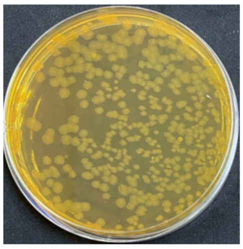	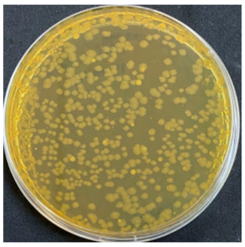
	Microbial load (CFU/mL)	3.24×10^{7}	3.56×10^{7}	3.60×10^{7}
P. aeruginosa	R (%)	-	-	-
			Time (min)	
		120	240	360
	Digital image	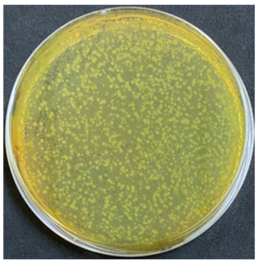	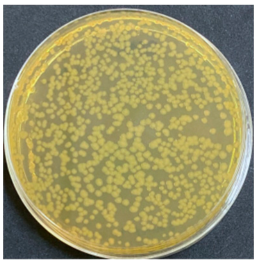	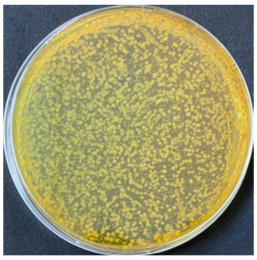
	Microbial load (CFU/mL)	5.71×10^{7}	5.75×10^{7}	5.82×10^{7}
	R (%)	-	-	-

R, the value of antimicrobial activity (%).

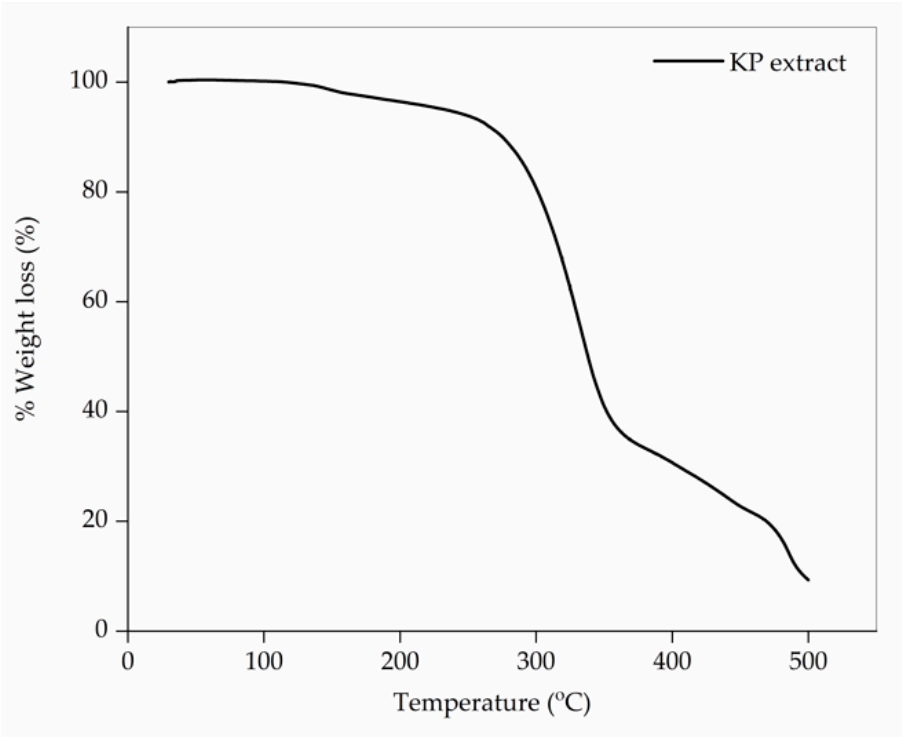

Figure S1. TGA thermogram of KP extract.

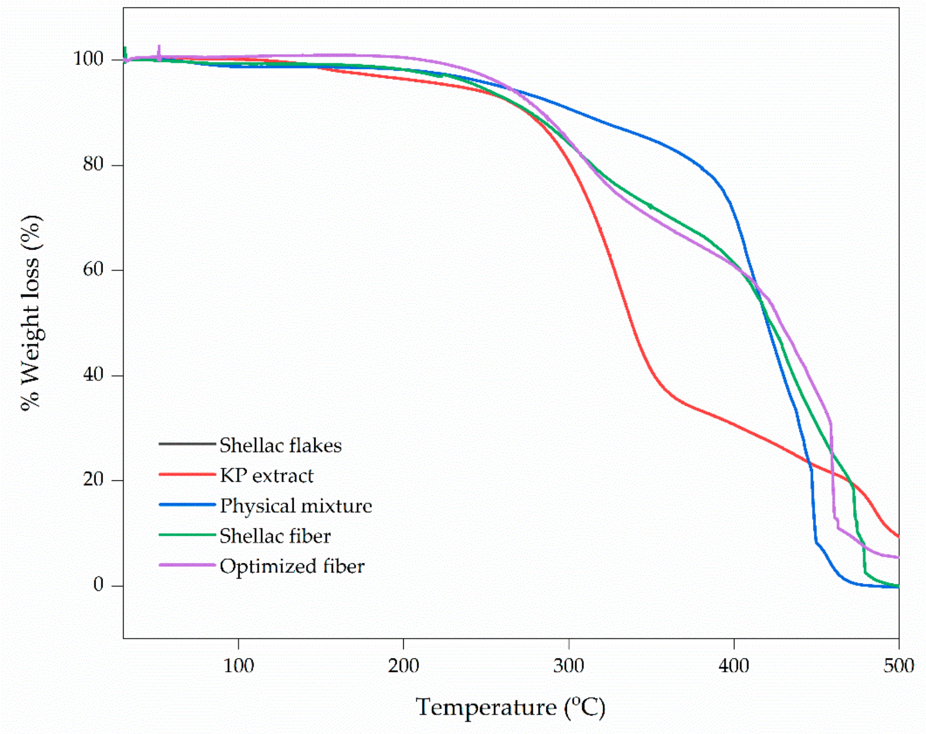

Figure S2. TGA thermograms of samples.

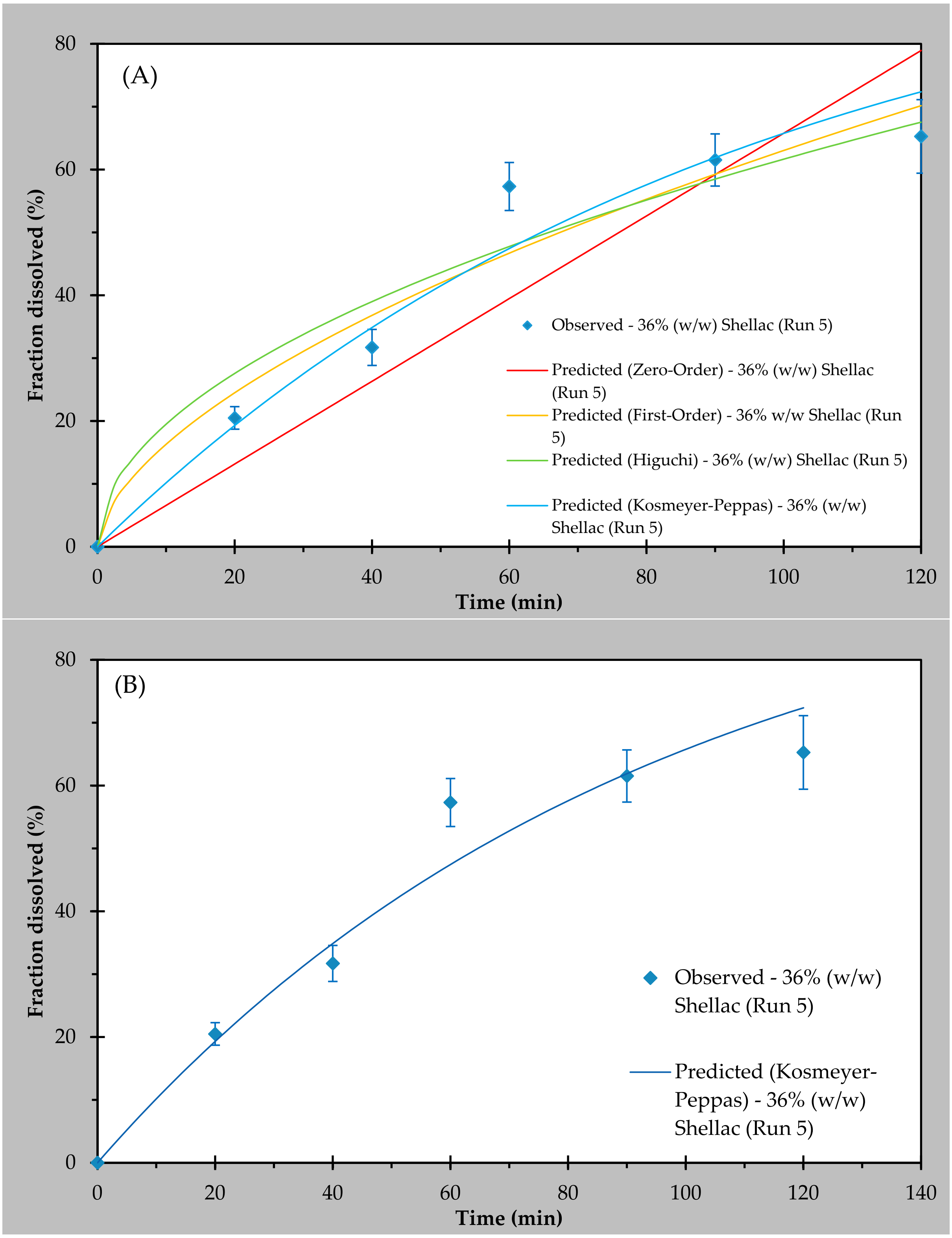

Figure S3. A mathematical model fits the KP extract release profiles of KP extract-loaded electrospun shellac fiber containing shellac and KP extract concentrations of 36% (*w/w*) and 3% (*w/w*), respectively: (A) All mathematical model fits, (B) The best fit model.

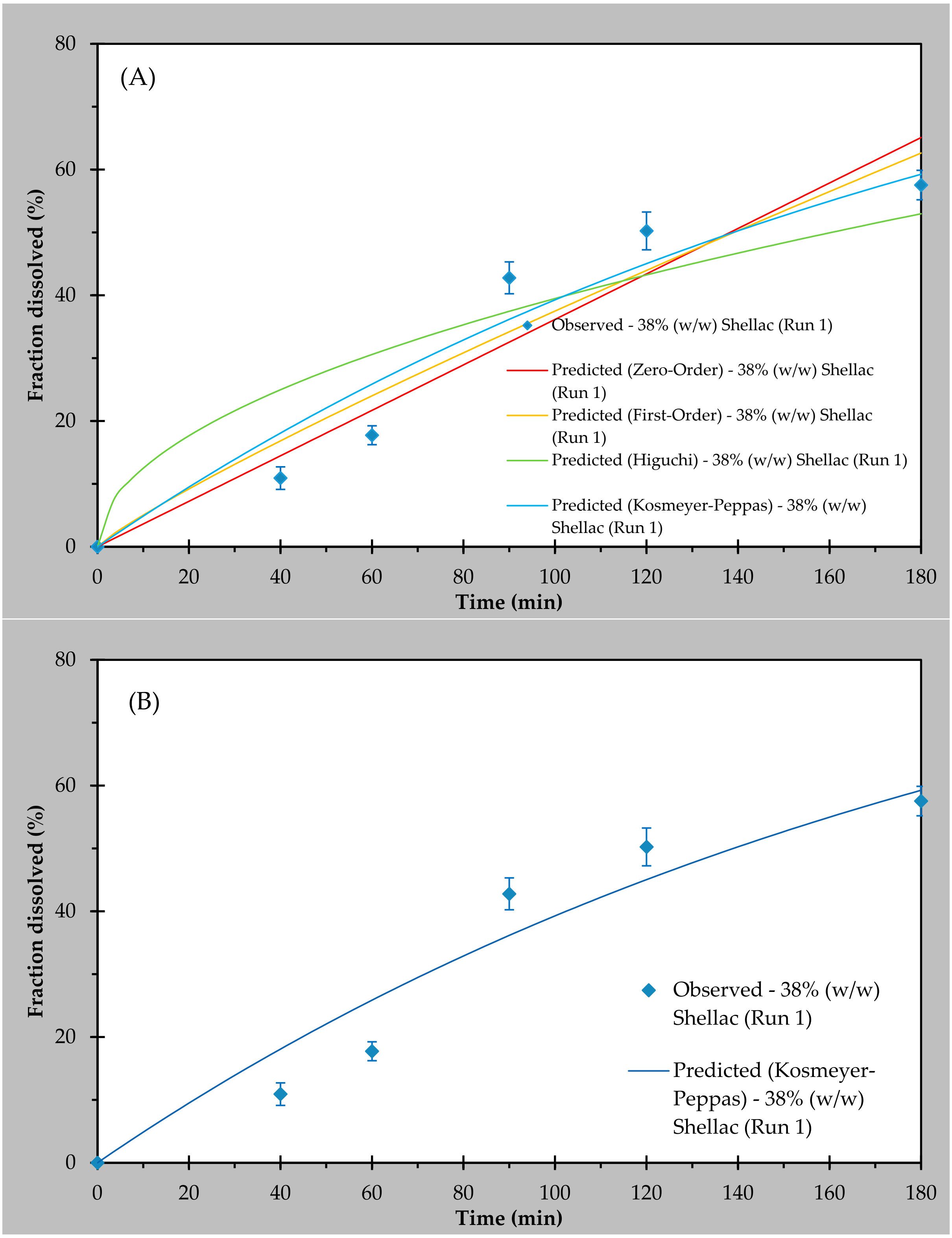

Figure S4. A mathematical model fits the KP extract release profiles of KP extract-loaded electrospun shellac fiber containing shellac and KP extract concentrations of 38% (*w/w*) and 3% (*w/w*), respectively: (A) All mathematical model fits, (B) The best fit model.

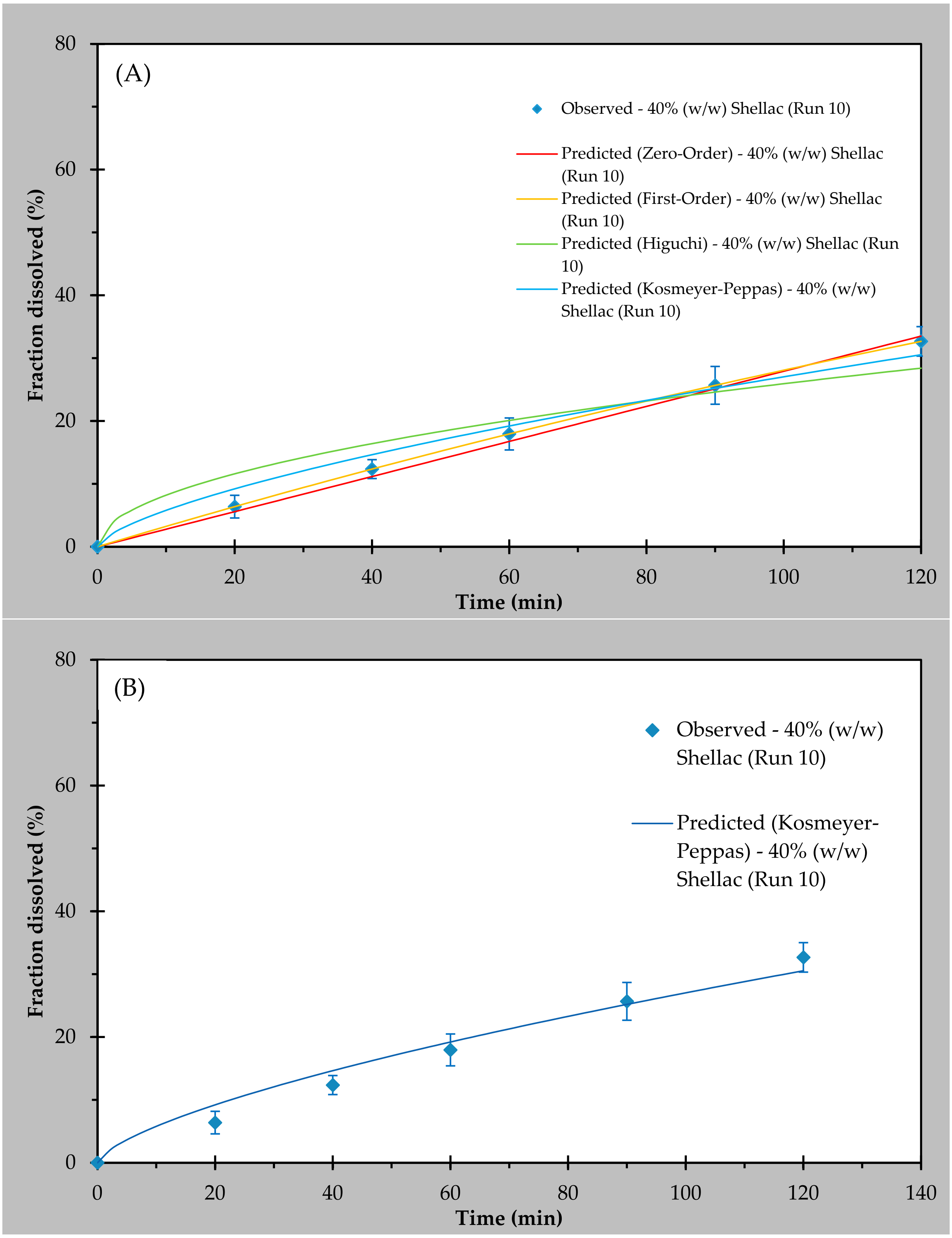

Figure S5. A mathematical model fits the KP extract release profiles of KP extract-loaded electrospun shellac fiber containing shellac and KP extract concentrations of 40% (*w/w*) and 3% (*w/w*), respectively: (A) All mathematical model fits, (B) The best fit model.

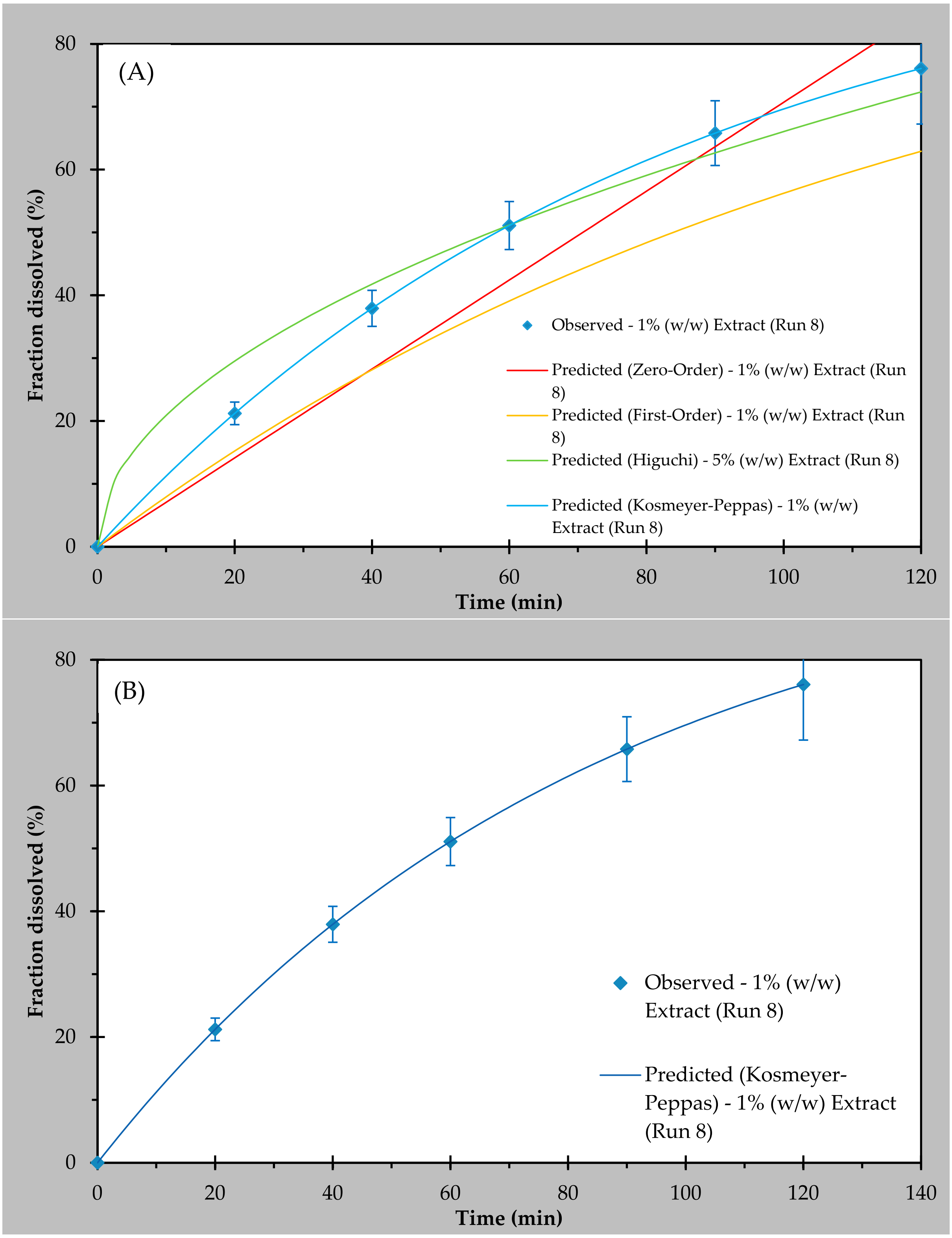

Figure S6. A mathematical model fits the KP extract release profiles of KP extract-loaded electrospun shellac fiber containing shellac and KP extract concentrations of 40% (*w/w*) and 1% (*w/w*), respectively: (A) All mathematical model fits, (B) The best fit model.

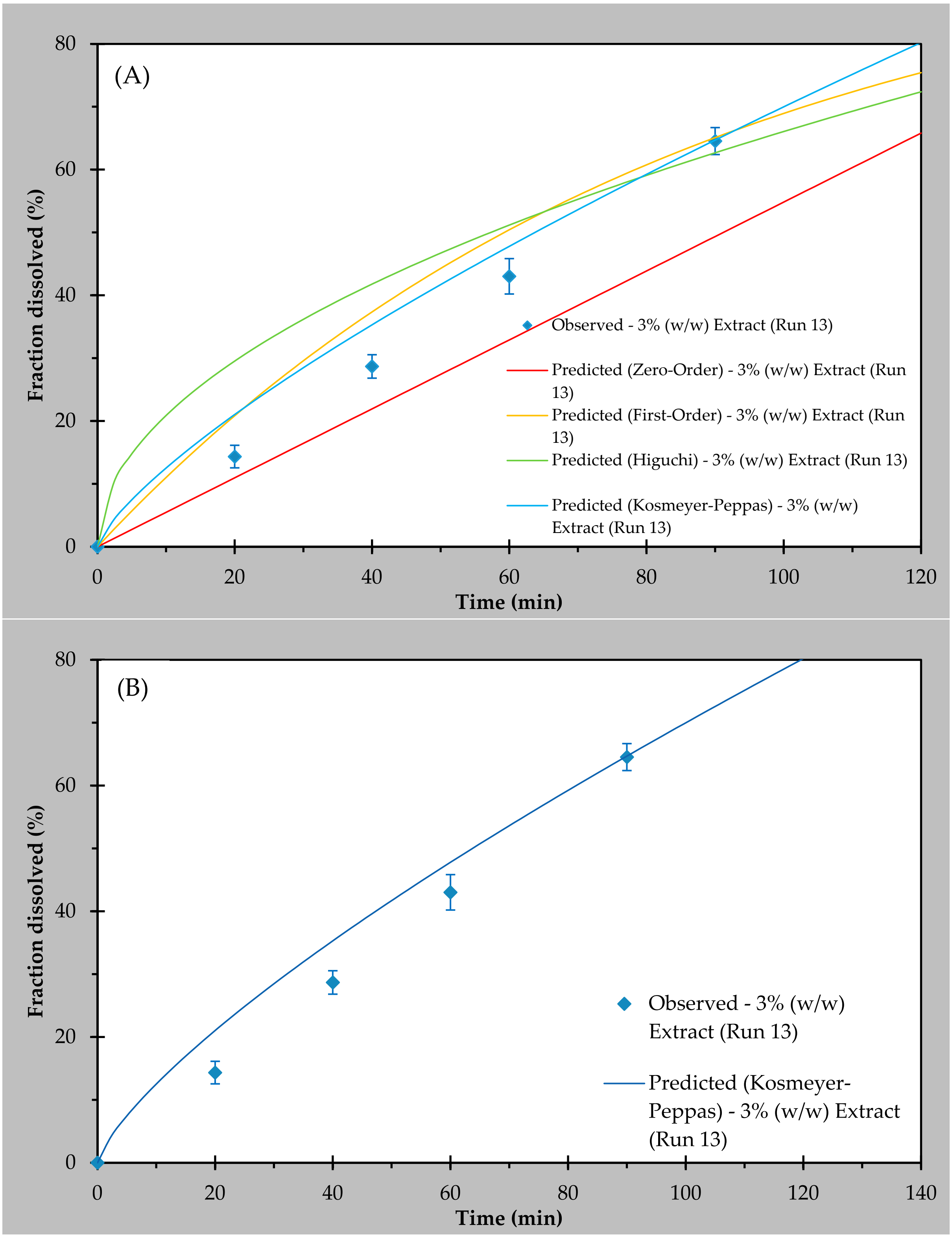

Figure S7. A mathematical model fits the KP extract release profiles of KP extract-loaded electrospun shellac fiber containing shellac and KP extract concentrations of 40% (*w/w*) and 3% (*w/w*), respectively: (A) All mathematical model fits, (B) The best fit model.

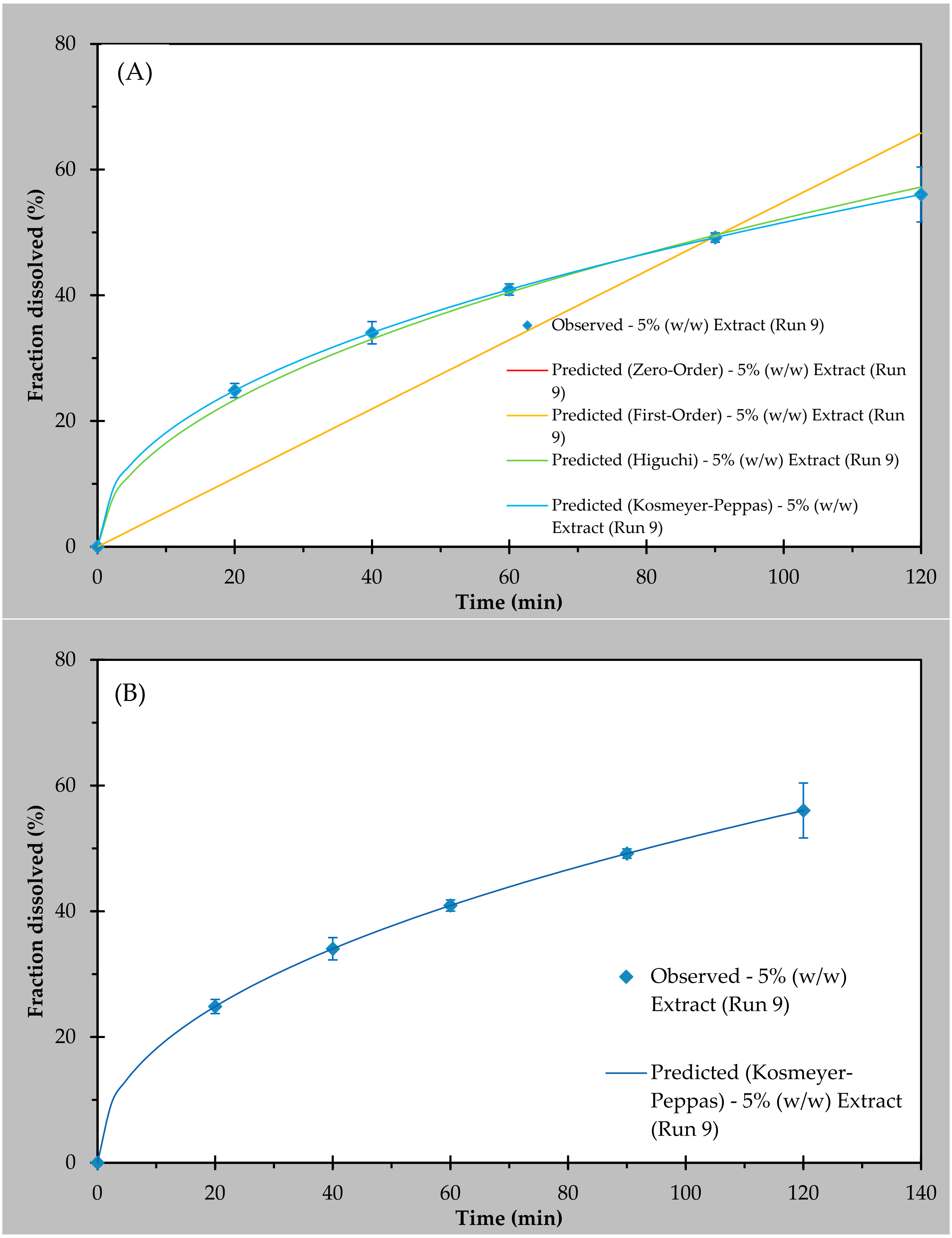

Figure S8. A mathematical model fits the KP extract release profiles of KP extract-loaded electrospun shellac fiber containing shellac and KP extract concentrations of 40% (*w/w*) and 5% (*w/w*), respectively: (A) All mathematical model fits, (B) The best fit model.

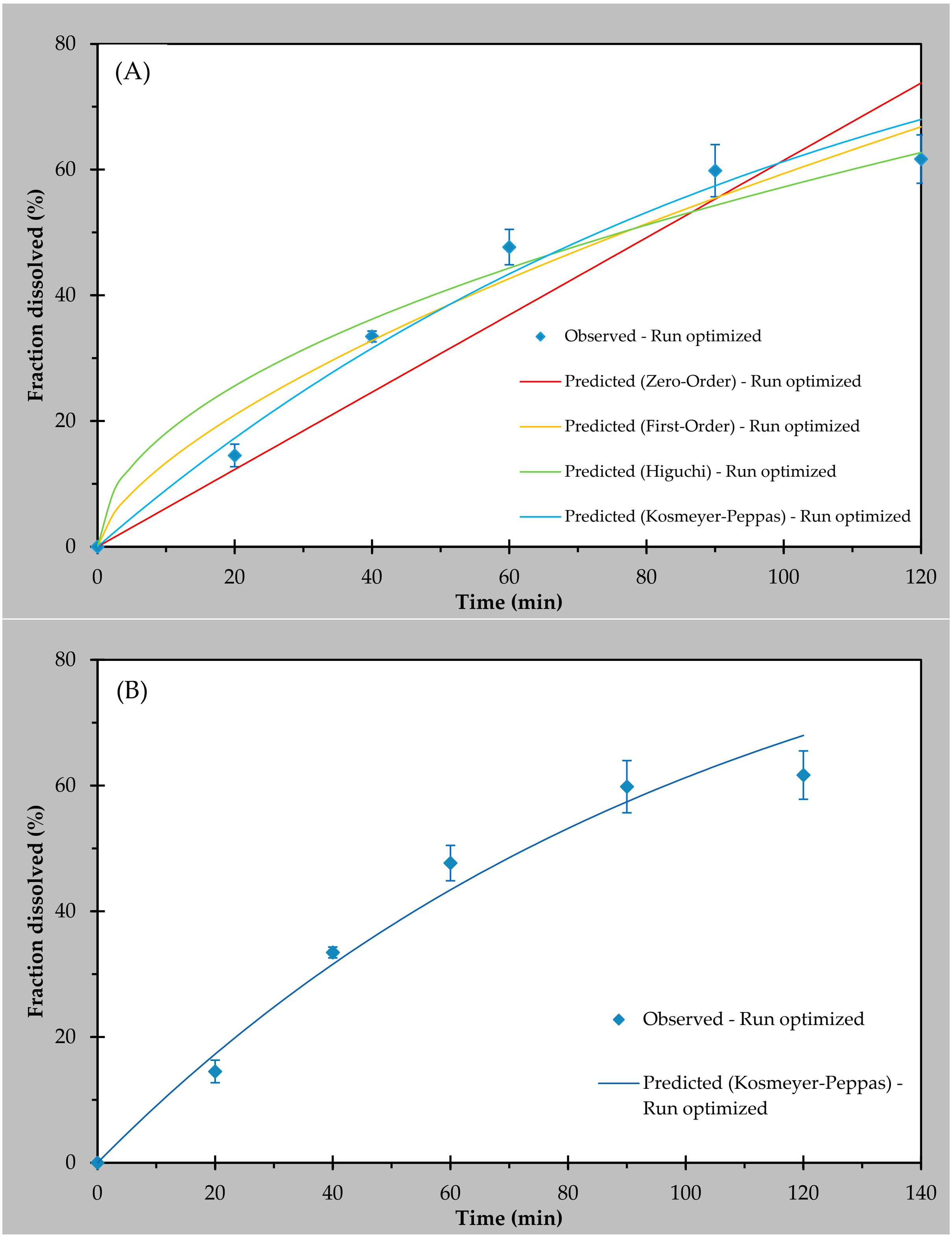

Figure S9. A mathematical model fits the KP extract release profiles of optimized KP extract-loaded electrospun shellac fiber containing shellac and KP extract concentrations of 37.25% (*w*/*w*) and 1.50% (*w*/*w*), respectively: (A) All mathematical model fits, (B) The best fit model.
